# Cardiac retransplantation is an efficacious therapy for primary cardiac allograft failure

**DOI:** 10.1186/1749-8090-3-26

**Published:** 2008-05-07

**Authors:** Pavan Atluri, William Hiesinger, Robert C Gorman, Alberto Pochettino, Mariell Jessup, Michael A Acker, Rohinton J Morris, Y Joseph Woo

**Affiliations:** 1 Division of Cardiovascular Surgery, Department of Surgery, University of Pennsylvania School of Medicine, Philadelphia, PA, USA; 2 Division of Cardiovascular Medicine, Department of Medicine, University of Pennsylvania School of Medicine, Philadelphia, PA, USA

## Abstract

**Background:**

Although orthotopic heart transplantation has been an effective treatment for end-stage heart failure, the incidence of allograft failure has increased, necessitating treatment options. Cardiac retransplantation remains the only viable long-term solution for end-stage cardiac allograft failure. Given the limited number of available donor hearts, the long term results of this treatment option need to be evaluated.

**Methods:**

709 heart transplants were performed over a 20 year period at our institution. Repeat cardiac transplantation was performed in 15 patients (2.1%). A retrospective analysis was performed to determine the efficacy of cardiac retransplantation. Variables investigated included: 1 yr and 5 yr survival, length of hospitalization, post-operative complications, allograft failure, recipient and donor demographics, renal function, allograft ischemic time, UNOS listing status, blood group, allograft rejection, and hemodynamic function.

**Results:**

Etiology of primary graft failure included transplant arteriopathy (n = 10), acute rejection (n = 3), hyperacute rejection (n = 1), and a post-transplant diagnosis of metastatic melanoma in the donor (n = 1). Mean age at retransplantation was 45.5 ± 9.7 years. 1 and 5 year survival for retransplantation were 86.6% and 71.4% respectively, as compared to 90.9% and 79.1% for primary transplantation. Mean ejection fraction was 67.3 ± 12.2% at a mean follow-up of 32.6 ± 18.5 mos post-retransplant; follow-up biopsy demonstrated either ISHLT grade 1A or 0 rejection (77.5 ± 95.7 mos post-transplant).

**Conclusion:**

Cardiac retransplantation is an efficacious treatment strategy for cardiac allograft failure.

## Background

The International Society for Heart Lung Transplantation (ISHLT) estimates that over 65,000 heart transplants have been performed worldwide. With the increasing population of heart transplant patients there is an associated large population of patients suffering from allograft failure resulting from 3 major categories: acute rejection, primary graft failure, and transplant arteriopathy. Several clinical and experimental strategies to treat graft failure have been proposed, but these options have not yet proven to be long-term clinical solutions. Cardiac retransplantation remains the only viable long-term treatment for end-stage cardiac allograft failure.

A few studies have evaluated outcomes following cardiac retransplantation and have demonstrated good results [1-4]. Unfortunately, the long-term efficacy of heart retransplantation remains unclear with several studies suggesting significantly worse outcomes [5-8]. Over the past two decades we have accumulated a significant degree of expertise in pre-, intra-, and post-operative management of heart transplant patients at our institution. Our results have been significantly better than the international mean. We undertook this retrospective study with the goal of evaluating cardiac retransplantation outcomes at a high volume center with good results. It is our hope that further evaluation of outcomes will elucidate the viability of orthotopic cardiac retransplantation as therapy for cardiac allograft failure.

## Methods

### Patient population

From April 1987 to May 2007 seven hundred and nine patients underwent orthotopic heart transplant for end-stage heart failure from various causes. Etiologies included ischemic heart disease (42.6%), dilated cardiomyopathy (55.6%), and other causes (1.8%). During this same period of time fifteen patients underwent repeat orthotopic heart transplantation.

Adverse factors, that could potentially limit long-term survival, were carefully examined and utilized as exclusion criteria included severe pulmonary hypertension, active or recent malignancy, evidence of end organ damage due to diabetes, major chronic disabling illness (e.g. lupus, severe arthritis, neurologic diseases, previous stroke with residual deficits), symptomatic peripheral vascular or carotid artery disease, active mental illness or psychosocial instability, HIV antibodies, intolerance of immunosuppression, and history of noncompliance. Only a fraction of patients felt to be suitable candidates for retransplantation received organs. Ultimately, whether a patient was retransplanted was determined primarily by organ availability and compatibility. Patients who were not candidates for retransplantation were not supported by ventricular assist devices.

At the University of Pennsylvania the average waiting time for heart transplant once listed with UNOS is 7.0 months. The majority of patients are transplanted within 1 year of listing (52.0%), while 15.2% of patients are transplanted within 30 days of listing. Only 5.7% of patients on the UNOS waiting list expire while awaiting transplantation at our institution (national average 13.4%).

The majority (50.0%) of patients were UNOS status 1A at the time of transplantation (14.3% – Status 1B, 35.7% – Status 2). The average survival of initial grafts, excluding hyperacute rejection (n = 1) and metastatic melanoma in the donor (n = 1) as the causes of retransplantation, was 62.1 ± 32.6 months. This study was performed utilizing existing data available in the University of Pennsylvania transplantation database along with clinical patient records.

### Operative strategy and patient management

Total ischemic time for allografts prior to reperfusion was 163 ± 29 minutes. 42.9% of all allograft anastomosis were performed in a bicaval fashion, the remainder were performed with a biatrial anastomosis. Average cardiopulmonary bypass time was 148 ± 48 minutes.

### Myocardial surveillance and pathologic analysis

Myocardial rejection was scored utilizing the ISHLT histopathologic grading nomenclature[9]. Myocardial surveillance biopsies were performed once every 2 weeks for the first 12 weeks, once a month for the remainder of the 1^st ^year, every six months during the 2^nd ^year, and then annually. Transthoracic echocardiography (TTE) was performed once a month for the 1^st ^3 months, every 6 months for the 1^st ^year, and then on an annual basis.

### Data analysis

Quantitative data are expressed as mean ± standard error of the mean (SEM). Variance between two groups was evaluated using the chi-square test. A *p*-value of less than 0.05 was considered statistically significant.

## Results

### Patient demographics

Cardiac retransplantation accounted for 2.1% of all heart transplants performed at our institution. Primary transplant allograft failure was due to transplant arteriopathy (66.7%), acute rejection (20.0%), hyperacute rejection (6.6%), and a post-transplant diagnosis of metastatic melanoma in the donor [10] (6.6%). 20.3% of recipients in the primary transplant group were women, as compared to 20% of recipients in the retransplant group. Mean age at retransplantation was 45.5 ± 9.7 years. Pertinent recipient variables are presented in Table 1. Mean donor age was 36.7 ± 9.9 years. One-third of all donors in this study were female.

**Table 1:** Retransplant recipient variables

Gender (female)	20%
Race (Caucasian)	89.4%
Initial Graft Survival (months)	62.1 ± 32.6
Blood Group	A (60%)
	B (6.7%)
	AB (0%)
	O (33.3%)
Recipient Body Mass Index (kg/m^2^)	27.9 ± 3.7
Pre-Operative Creatinine (mg/dl)	2.5 ± 1.5
Chronic Renal Insufficiency (Cr>1.5)	60.0%
Diabetes Mellitus	20.0%
Hypertension	66.7%
Hypercholesterolemia	53.3%

One patient in this study was supported on extracorporeal membrane oxygenation (ECMO) for 24 hours, secondary to hyperacute rejection, while awaiting retransplantation. There were no untoward sequallae following ECMO. None of the patients were supported on ventricular assist devices (VAD).

### Post-operative management

Post-operatively patients were recovered in the cardiothoracic surgical intensive care unit (CT SICU) until they were hemodynamically stable for transfer to the cardiothoracic step-down unit. Mean post-operative cardiac index was 3.4 ± 0.7 l/min/m^2^. On average patients remained intubated for only 8.6 ± 4.3 hours and remained in the CT SICU for 3.5 ± 1.0 days. Average length of hospital stay was 13.2 ± 4.5 days. Patients remained in the hospital in order to undergo surveillance myocardial biopsies prior to discharge.

Transthoracic echocardiography was performed 24 hours following transplantation. Mean ejection fraction immediately following transplant was 65.6 ± 14.2%. Three patients demonstrated moderately decreased left ventricular function which resolved prior to hospital discharge without the assistance of pressors. Two patients had mild tricuspid regurgitation (TR) while an additional two patients demonstrated moderate TR. Three out of four patients with tricuspid regurgitation underwent biatrial anastomosis, while the remaining patient with moderate TR had bicaval anastomosis. Mean ejection fraction on discharge was improved to 68.9 ± 6.8%. All patients underwent percutaneous endomyocardial biopsies prior to discharge. Ten patients demonstrated no histopathologic evidence of rejection. Four patients had mild rejection (ISHLT Grade 1), while 1 patient had moderate rejection (ISHLT Grade 3A).

There were no major hemodynamic complications following retransplantation necessitating operative reexploration. One patient suffered a mild, non-debilitating cerebrovascular event without residual deficit. Another patient required a right sided thoracostomy tube for a hemothorax. Two patients required hemodialysis for a short period of time immediately following transplantation, but regained adequate renal function prior to discharge. Mean 24 hour chest tube output was 525 ± 202 cc. On average patients were transfused with 3.3 ± 1.6 units of packed red-blood cells.

### Long term patient survival

Patient survival following primary heart transplant at our institution is 90.9% 1 year following transplant and 79.1% 5 years following transplant. This is better than the international average heart transplant survival as published by the ISHLT for primary cardiac transplantation with survivals of roughly 85% at 1 year and 70% at 5 years[11]. Patients in this study undergoing cardiac retransplantation demonstrated 1- and 5-year survivals of 86.6% (p = NS, compared to primary transplants) and 71.4% (p = NS) respectively. Mean ejection fraction was 67.3 ± 12.2% at a mean follow-up of 32.6 ± 18.5 mos post-retransplan; follow-up biopsy demonstrated either ISHLT grade 1A or 0 rejection (77.5 ± 95.7 mos post-transplant).

Only 1 patient expired due to failure of the retransplanted allograft. One day following retransplantation a patient expired secondary to overwhelming fungal sepsis resulting from presumed colonic necrosis. There was 1 late death 9 months following retransplant resulting from micronodular cirrhosis diagnosed 4 months following transplant. Lastly, a patient expired 27 months following retransplantation due to metastatic lung cancer.

## Discussion

As demonstrated in this study, cardiac retransplantation can be safely performed in appropriately selected patients with good outcomes. We have demonstrated long term viability following cardiac retransplantation that approaches that of primary cardiac transplantation. With appropriate patient selection, meticulous intra- and post-operative care, and careful myocardial surveillance cardiac retransplantation is a very effective and viable treatment strategy.

Alternate therapeutic options for cardiac allograft failure are limited as minimal benefits have been demonstrated with percutaneous coronary interventions, coronary artery bypass grafting, valvular repair, plasmapheresis, or medical management [12-15]. There are several promising experimental strategies, including myocardial regeneration and vasculogenesis that may provide novel and minimally invasive means of salvaging failing, end-stage cardiac allografts [16-19]. To date, however, these therapies remain experimental and are beyond the current clinical scope. Therefore, the only viable therapy for end-stage heart failure following a cardiac transplant remains cardiac retransplantation. Roughly 2.0% of all heart transplants performed annually are cardiac retransplants[11].

The literature on cardiac retransplantation is ambiguous with several studies reporting divergent findings. Shuhaiber and colleagues in a retrospective review evaluating cardiac retransplants found a higher risk of death after retransplantation when compared to primary allograft failure. Risk factors for cardiac retransplantation were ischemic time, age, and ventilator dependence at the time of transplant, these were similar risk factors for cardiac transplantation[6]. Optimizing these risk factors with appropriate patient selection can significantly improve retransplantation outcomes. Similarly, a smaller study by Schnetzler and colleagues demonstrated 1- and 4-year survivals of 61.5% and 46% respectively. Interestingly, the initial transplant patients in this study had 1 year survival rates close to 70%, a finding that is significantly lower than the 85% international average reported by the ISHLT registry[11]; thereby putting in to question the overall comparability of the data.

A high volume transplant center published a retrospective review of their retransplant experience and reported slightly lower but beneficial short- and long-term results following cardiac retransplantation[8]. This finding was corroborated by both a multi-institutional study and single center experience that found cardiac retransplantation to be a viable option for allograft failure secondary to vasculopathy[1,3]. Our findings in this study, from a high volume center with extensive heart transplant experience and very good long-term results, further support cardiac retransplantation for allograft failure following a heart transplant. Admittedly this study is limited by the small number of patients and retrospective analysis of the data. Even with this limitation, we have extracted valuable outcomes that support relisting appropriate patients for cardiac retransplantation. A large prospective study is warranted and should be performed, but given the small number of cardiac retransplantations performed internationally a study of this magnitude would take several years to complete.

From a theoretical and immunologic standpoint, retransplantation in a patient with long standing immunosuppression may actually provide the appropriate milieu for diminished rejection, decreased coronary vasculopathy, and longer allograft survival. One of the most controversial post-transplant issues has been the utilization of induction therapy with cytolytic anti-lymphocyte antibodies such as OKT3 and thymoglobulin. Encouragingly, induction therapy has been associated with decreased rates of allograft rejection[20,21]. But, on the other hand, utilization of anti-lymphocyte antibodies has been associated with 9 fold higher rates of lymphoproliferative disorders, bacterial and viral infections, meningitis, and respiratory distress[22,23]. Based, on this information one could surmise that a pre-existing immunosuppressed status would potentiate the beneficial decrease in rejection seen with induction therapy while avoiding the negative side effects associated with cytolytic antibodies. If this theory holds true, then retransplantation from a purely immunological standpoint would confer greater long term graft viability.

Along similar lines retransplantation in patients with prolonged initial graft survival, ie. greater than 15 years, will likely have longer retransplant graft function as compared to patients who suffer hyperacute rejection or repeated bouts of rejection and rapid onset allograft dysfunction. Rapid rejection implies a profound immunologic response with less tolerance of allogenic grafts. A large, multi-institutional retrospective study found worse survival in patients undergoing cardiac retransplantation for early graft failure and acute rejection; with survival rates for patients that underwent retransplantation <6 months after initial transplant manifesting worse survival[3]. Therefore, retranplantation performed in a semi-elective setting for transplant arteriopathy will likely confer better results than emergent retransplantation performed for acute rejection. Recipient age does not appear to affect long-term survival[3].

Cardiac retransplantation raises further interesting management questions. For instance, does it matter if we do not resect all of the previous heart? The renal transplant literature suggests that the presence of multiple organs from different donors increases the risk of organ rejection. One can theorize that this situation would hold true for cardiac retransplants with residual tissue from the initial donor as well as a heart from a second donor. Therefore, if a patient has had a biatrial anastomosis at the initial transplant, should all of the initial donor aorta, pulmonary artery, and atria be resected to decrease immunogenicity and rejection associated with the "chimera"? The literature in both clinical and experimental cardiac transplant models thus far fails to answer these issues. These questions exemplify our basic understanding of cardiac retransplantation and the need to expand our knowledge so that we can optimize outcomes.

## Conclusion

In conclusion, cardiac retransplantation is an efficacious treatment strategy for cardiac allograft failure with good long-term results.

## Competing interests

The authors declare that they have no competing interests.
